# Vitamin D Inhibits IL-6 Pro-Atherothrombotic Effects in Human Endothelial Cells: A Potential Mechanism for Protection against COVID-19 Infection?

**DOI:** 10.3390/jcdd9010027

**Published:** 2022-01-13

**Authors:** Giovanni Cimmino, Stefano Conte, Mariarosaria Morello, Grazia Pellegrino, Laura Marra, Andrea Morello, Giuseppe Nicoletti, Gennaro De Rosa, Paolo Golino, Plinio Cirillo

**Affiliations:** 1 Department of Translational Medical Sciences, Section of Cardiology, University of Campania “Luigi Vanvitelli”, 80131 Naples, Italy; giovanni.cimmino@unicampania.it (G.C.); paolo.golino@unicampania.it (P.G.); 2 Department of Translational Medical Sciences, Section of Lung Disease, University of Campania “Luigi Vanvitelli”, 80131 Naples, Italy; stefanoconte86@gmail.com; 3 Department of Advanced Biomedical Sciences, Section of Cardiology, University of Naples “Federico II”, 80131 Naples, Italy; mariarosaria.morello@gmail.com (M.M.); giuseppenicoletti1992@gmail.com (G.N.); gderosa@unina.it (G.D.R.); 4 Department of Woman, Child and General and Specialized Surgery, Section of Anesthesiology, University of Campania “Luigi Vanvitelli”, 80138 Naples, Italy; stella.graziap@gmail.com; 5 Department of Cell Biology and Biotherapy Research, Istituto Nazionale Tumori IRCCS—Fondazione G. Pascale, 80131 Naples, Italy; laurama85@gmail.com; 6 Biochemical Unit, A. S. Re. M. (Azienda Sanitaria Regionale del Molise), Antonio Cardarelli Hospital, 86100 Campobasso, Italy; dr_andrea.morello@hotmail.it

**Keywords:** atherothrombosis, COVID-19, IL-6, tissue factor, vitamin D

## Abstract

Background: Thrombosis with cardiovascular involvement is a crucial complication in COVID-19 infection. COVID-19 infects the host by the angiotensin converting enzyme-2 receptor (ACE2r), which is expressed in endothelial cells too. Thus, COVID-related thrombotic events might be due to endothelial dysfunction. IL-6 is one of the main cytokines involved in the COVID-19 inflammatory storm. Some evidence indicates that Vitamin D (VitD) has a protective role in COVID-19 patients, but the molecular mechanisms involved are still debated. Thus, we investigated the effect of VitD on Tissue Factor and adhesion molecules (CAMs) in IL-6-stimulated endothelial cells (HUVEC). Moreover, we evaluated levels of the ACE2r gene and proteins. Finally, we studied the modulation of NF-kB and STAT3 pathways. Methods: HUVEC cultivated in VitD-enriched medium were stimulated with IL-6 (0.5 ng/mL). The TF gene (RT-PCR), protein (Western blot), surface expression (FACS) and procoagulant activity (FXa generation assay) were measured. Similarly, CAMs soluble values (ELISA) and ACE2r (RT-PCR and Western blot) levels were assessed. NF-kB and STAT3 modulation (Western blot) were also investigated. Results: VitD significantly reduced TF expression at both gene and protein levels as well as TF-procoagulant activity in IL-6-treated HUVEC. Similar effects were observed for CAMs and ACE2r expression. IL-6 modulates these effects by regulating NF-κB and STAT3 pathways. Conclusions: IL-6 induces endothelial dysfunction with TF and CAMs expression via upregulation of ACE2r. VitD prevented these IL-6 deleterious effects. Thus, it might be speculated that this is one of the hypothetical mechanism(s) by which VitD exerts its beneficial effects in COVID-19 infection.

## 1. Introduction

Inflammation with associated thrombosis known as “thrombo-inflammation” is a common feature of several human diseases [1]. Thrombo-inflammation occurs during sepsis, ischemia-reperfusion injury, organ transplant rejection, major trauma, severe burns, antiphospholipid syndrome, preeclampsia, sickle cell disease, and biomaterial-induced thrombo-inflammation [1]. In thrombo-inflammation, endothelium loses its physiological anti-thrombotic and anti-atherosclerotic functions [2,3]. Specifically, dysfunctional endothelium turns to a pro-atherothrombotic phenotype by expressing adhesion molecules such as VCAM/ICAM, actively involved in pathophysiology of atherosclerosis [4] and Tissue Factor (TF) [3], the key initiator of coagulation cascade [5]. Interestingly, thrombo-inflammation seems to be also a main feature of the recent novel COVID-19 infection [6]. This virus, besides causing lung failure, seems responsible for severe cardiovascular complications. Specifically, many vascular thrombotic events including acute coronary syndromes have been observed, representing a rationale for antithrombotic therapy in COVID-19 patients [7]. COVID-19 infects the host by binding to the angiotensin converting enzyme 2 receptor (ACE2r) [8]. This receptor is widely expressed in the lung, which represents the main virus target, but it has been isolated also in the heart, kidney, and intestine [9]. In patients with COVID-19 infection, an uncontrolled inflammatory response with release of several cytokines has been observed [10,11,12]. In this cytokine storm, IL-6 seems to play an important role [10] witnessed also by a relationship between higher IL-6 levels, alveolar damage, extrapulmonary injury and increased mortality [11,12].

Some evidence has suggested that Vitamin D (VitD) might exert its biological effects in many other systems apart from musculoskeletal tissues since lower plasma levels of this vitamin have been associated with infectious and autoimmune diseases, cancer and cardiovascular diseases [13]. Indeed, some studies have suggested a hypothetical link between VitD deficiency and the development of cardiovascular as well as of respiratory viral infections [13,14,15,16]. To date, it has been reported that lower levels of VitD might be associated with a worse outcome in patients with COVID-19 infection too [17]. Although all this evidence has suggested a protective role from VitD in these diseases, the mechanisms by which this vitamin might exert its beneficial effects have not been completely elucidated yet. Thus, starting from this scientific background, we studied the effects of VitD on human endothelial cells incubated with IL-6. Moreover, we investigated some mechanisms potentially involved in modulating these VitD-mediated phenomena.

## 2. Materials and Methods

### 2.1. Cell Cultures

Human umbilical vein endothelial cells (HUVECs) were purchased from Lonza (Basel, Switzerland). Cells were grown in EGM 2 medium with 10% FBS (Sigma Chemical Co., St Louis, MO, USA), and used at passages 2 to 5. They were enzymatically harvested and counted in a haemocytometer and subcultured in 24-well plates at an initial density of about 5 × 104 cell/well or 100 mm cell plates at the density of about 2 × 10^6^ cells per plate according the experimental protocol. At confluence, cells were starved in serum-free medium for 24 h and then used in the different set of experiments. All reagents, media and water used for the experiments were tested for possible endotoxin contamination by Limulus assay (Bio Whittaker, Walkersville, MD, USA). An endotoxin level <0.125 EU/mL was found.

### 2.2. Experimental Protocol

At confluence, cells were starved in serum-free medium for 24 h enriched with VitD (1a,25-dihydroxyvitamin D3, Catalog Number D1530, Sigma-Aldrich, St. Louis, MO, USA; dissolved in ethanol 0.1% following manufacturer’s instruction), at final concentration of 10 nM for 1 h, as already reported [18,19], and then incubated with native IL-6 (0.5 ng/mL). LPS-stimulated cells (50 µg/mL) served as positive control and non-VitD treated cells served as negative control. Evaluation of Tissue Factor expression was assessed as previously reported [19] at 1 and 2 h for gene and 6 and 12 h for protein levels. Surface expression and activity were assessed at 6 h. Similarly, ACE2r, Caspase-1 (as main part of the Inflammasome) and CAMs expression were evaluated. Finally, to verify the effective involvement of VitD receptor (VDR) in the action mechanism of VitD, HUVECs were also treated with VDR antagonist ZK159222 (Bayer Schering Pharma AG, Berlin, Germany). This VDR antagonist was used at the same concentration as VitD (10 nM) as already reported [19]. Expression of TF-mRNA and procoagulant activity were evaluated.

#### 2.2.1. Tissue Factor Gene and Protein Levels, Surface Translocation and Functional Activity

Tissue Factor gene levels were evaluated in stimulated HUVECs and were assessed at 1 and 2 h as previously described [19]. After stimulation, cells were washed with phosphate-buffered saline (PBS). Total mRNA was extracted using TRIzol reagent (GIBCO, Carlsbad, CA, USA), according to standard protocol. Reverse transcription was performed using SensiFAST cDNA (BIOLINE) and 100 ng of the RNA samples from each culture condition. Samples were run in triplicate in 20 μL reactions by using a Rotor-Gene Q sequence detector system (QIAGEN). Samples were incubated at 95 °C for 2 min and then underwent 40 cycles at 95 °C for 5 s and 60 °C for 1 min using SYBR green chemistry (SensiFAST SYBR, BIOLINE). Specific oligonucleotides for human GAPDH and human TF were designed using PRIMER EXPRESS Software (Applied Biosystems, Waltham, MA, USA ) on the basis of published sequences [19]. The results were analyzed using a comparative method, and the values were normalized to the GAPDH expression and converted into percentage change. Three different experiments were performed for each experimental condition.

Tissue Factor protein levels were performed in cell lysates at 6 and 12 h following IL-6 stimulation. The samples (30 µg) were treated with SDS-PAGE sample buffer, followed by heating, and then subjected to 10% gel. The protein was transferred onto membranes with an iBLOT2 Dry Blotting System (Invitrogen, Waltham, MA, USA), according to the manufacturer’s instructions. TF was detected with a specific antibody (1:1000, American Diagnostica Inc., Greenwich, CT, USA). After detecting TF, the membranes were stripped and then treated to detect tubulin expression as housekeeping protein. Band intensities were quantified using Image 1 J software (densitometric units × 10^3^) and graphically expressed. Six different experiments were performed for each experimental condition.

Membrane expression of TF was assessed at 6 h by FACS analysis after IL-6 incubation. For this analysis, HUVECs were detached with 10 mmol/L EDTA in PBS (without trypsin) and stained with FITC-labelled monoclonal antibody (Pharmingen, Franklin Lakes, NJ, USA) against TF, or with the appropriate isotype IgG (phycoerythrin or FITC) as control. Fluorescence intensity of 9000 cells for each sample was quantified by a FACSCalibur analyzer (Becton-Dickinson, Franklin Lakes, NJ, USA). Functional activity of the IL-6-induced TF was evaluated using a specific assay colorimetric assay, based on the ability of TF to promote generation of FXa, as previously described [19,20]. Briefly, after IL-6 stimulation, cells were washed and incubated with 1 nM of recombinant human FVIIa (Novo Nordisk A/S, Gentofte, Denmark), followed by 100 nM of purified human FX (Calbiochem Novobiochem, La Jolla, CA, USA) and 5 mM CaCl_2_ for 15 min at 37 °C. A chromogenic substrate, specific for FX (Cromozym X, Roche Diagnostics, Mannheim, Germany, 0.5 mmol/L) was then added and incubated for 30 min at 37 °C. The reaction was stopped by adding 200 µL/mL of acetic acid (30% solution), and the change in optical density at 405 nm was quantified with a spectrophotometer. Standard curve was generated using purified FXa of known concentration (Sigma Chemical Co., St Louis, MO, USA). Six different experiments were performed for each experimental condition.

#### 2.2.2. Evaluation of ACE2 Receptor-mRNA and Protein Levels

Gene expression of ACE2 receptor was evaluated by Real Time PCR. Endothelial cells were incubated with IL-6 and ACE2r-mRNA levels were measured at 4 h. Specific oligonucleotides for human ACE2r (FW: 5′-GGGATCAGAGATCGGAAGAAGAAA-3′, REV: 5-AGGAGGTCTGAACATCATCAGTG-3′) were designed on the basis of published sequences using PRIMER EXPRESS Software (Applied Biosystems, Waltham, MA, USA) and validated for their specificity. The results were analyzed using a comparative method, and the values were normalized to the GAPDH expression and converted into percentage change. Three different experiments were performed for each experimental condition.

ACE2r protein levels were measured in IL-6-stimulated HUVECs by Western Blot analysis according to standard protocol as indicated. ACE2r was detected at 8 h after stimulation with a specific monoclonal antibody (Invitrogen MA5-26629). After detecting ACE2r, the membranes were stripped and then treated to detect tubulin expression as housekeeping protein. Band intensities were quantified using Image 1 J software (densitometric units × 10^3^) and graphically expressed. Six different experiments were performed for each experimental condition.

#### 2.2.3. Effects on Adhesion Molecules Expression

Soluble ICAM-1 and VCAM-1 levels were measured in HUVECs at 12 h upon IL-6 stimulation using specific ELISA assays (BMS201 and KHT0601, respectively, purchased from Thermo Fisher Scientific, Waltham, MA, USA). Both assays were run according manufacturer’s instructions. Six different experiments were performed for each experimental condition [19].

#### 2.2.4. Regulation of Intracellular Signaling: The Role of NF-κB Nuclear Translocation and STAT3

It has been reported that IL-6 activates several intracellular pathways, such as NF-κB and STAT3 [21] and that these pathways up-regulate TF and CAMs expression. Thus, the potential involvement of these intracellular pathways has been investigated in our experimental protocol. For this purpose, HUVECs were incubated with IL-6 as above for 4 h and then NF-κB translocation was evaluated by using a non-radioactive, sensitive method for detecting the specific transcription factor DNA binding activity (Cayman Chemical, Ann Arbor, MI, USA). STAT3 levels were measured by an ELISA kit EKC2516 (Boster Bio, Pleasanton, CA, USA). In parallel, IκB protein levels were assessed by Western blot analysis using an anti-IκB antibody (1:1000 dilution purchased from Thermo Fisher Scientific, Waltham, MA, USA). Three different experiments were performed for each experimental condition.

#### 2.2.5. Modulation of NLRP3 Protein

Some evidence has shown that the cytokine storm with elevated levels of IL-1 and IL-6 observed in SARS-CoV2 patients is due to the activation of NLRP3 inflammasome [22,23]. Thus, in an additional set of experiments we studied the effect of VitD on Caspase-1, a component of the active inflammosome. HUVECs were treated as above. Caspase-1 levels were measured at 6 h by Western blot using Caspase-1 p20 antibody orb221355 (Biorbyt, Cambridge, UK). After detecting Caspase-1, the membranes were stripped and then treated to detect tubulin expression as housekeeping protein. Band intensities were quantified using Image 1 J software (densitometric units × 10^3^) and graphically expressed. Six different experiments were performed for each experimental condition.

### 2.3. Statistical Analysis

Data are presented as mean ± SD. Differences between groups were determined by a one way ANOVA followed by a Student’s t test with Bonferroni’s correction. A *p* value < 0.05 was considered statistically significant. In all statistical analysis, SPSS 22.0 Statistical Package Program for Windows (SPSS Inc., Chicago, IL, USA) was used.

## 3. Results

### 3.1. Tissue Factor Gene and Protein Levels, Surface Translocation and Procoagulant Activity

**Figure 1:**
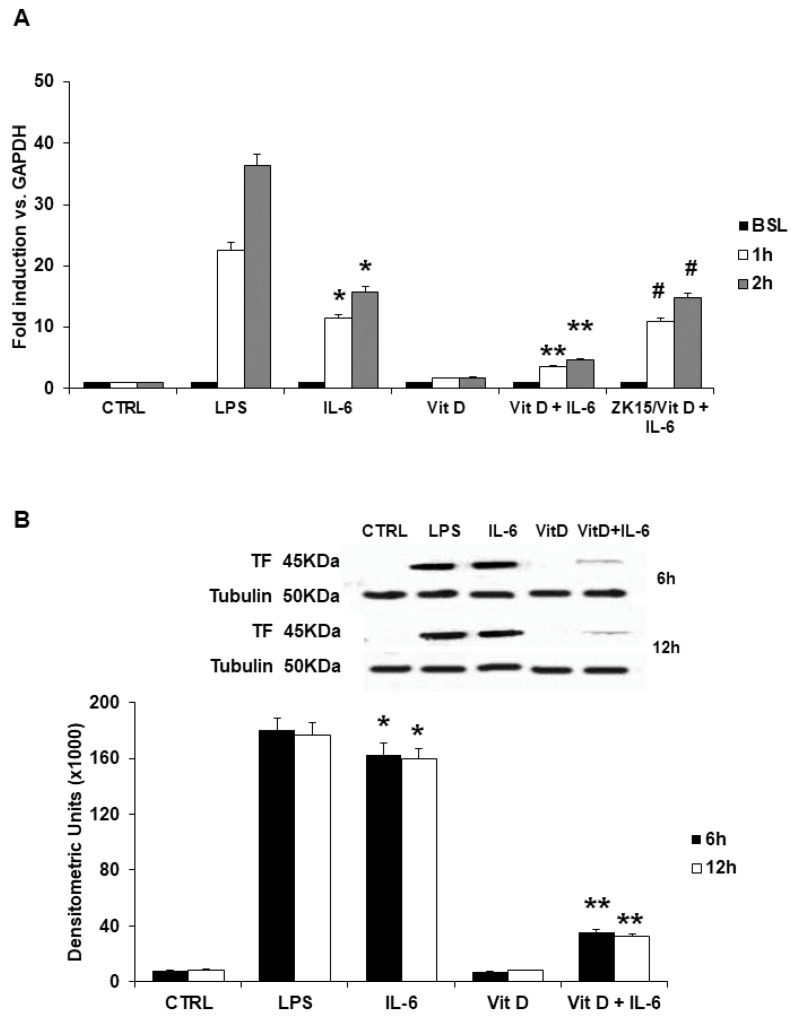
(**A**) Effects of VitD on IL-6-induced TF transcription in human endothelial cells assessed by Real Time quantitative PCR. TF mRNA was undetectable at baseline. Incubation with IL-6 caused significant increase in TF-mRNA levels, as compared to unstimulated cells. Preincubation with VitD inhibited the IL-6 effect on TF-mRNA. The use of ZK15, a VDR antagonist, completely abolished its effect. LPS served as positive control. Data are expressed as fold induction versus control gene represented by GAPDH. Each bar represents the mean ± SD of 3 different experiments. (* *p* < 0.001 vs. Control; ** *p* < 0.001 vs. IL-6; ^#^ *p* = NS vs. IL-6; one-way ANOVA with Tukey’s post hoc test). (**B**) Effects of VitD on IL-6-induced TF protein evaluated by Western blot analysis of cell lysates. IL-6 caused a significant increase in TF protein levels. These effects were prevented by treatment with VitD. Tubulin served as loading control. LPS served as positive control. Each bar represents the mean ± SD of 6 different experiments (* *p* < 0.001 vs. Control; ** *p* < 0.001 vs. IL-6; one-way ANOVA with Tukey’s post hoc test). The insert shows results of a representative experiment.

In line with previous reports [19,20], TF gene levels were almost undetectable in unstimulated endothelial cells. Incubation with IL-6 caused a significant increase in TF-mRNA levels in a time-dependent manner as compared to unstimulated cells (Figure 1A). The increased effect of LPS as positive control is also shown. These IL-6-mediated effects were significantly reduced in HUVECs incubated with VitD (Figure 1A). Moreover, incubation with a specific VDR antagonist (ZK15), completely abolished the reduction of TF gene levels exerted by VitD (Figure 1A). Similarly, TF protein levels assessed by Western blot, were almost undetectable in unstimulated HUVECs. Incubation with IL-6 induced TF protein expression in a time-dependent manner, which was significantly lower in cells preincubated with VitD (Figure 1B).

**Figure 2:**
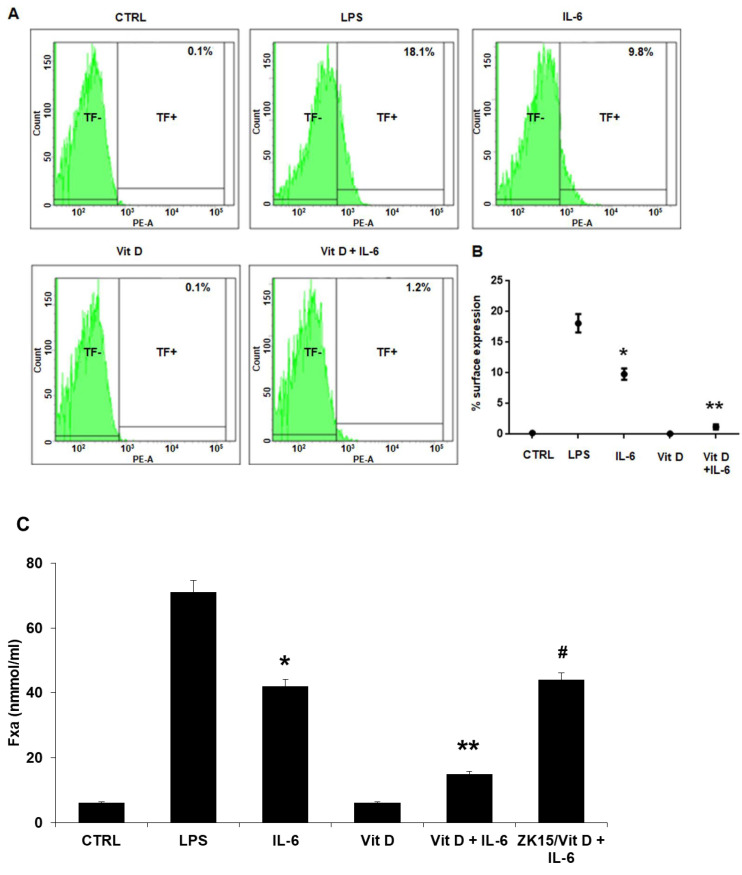
(**A**) FACS analysis showed that IL-6 induced TF expression on cell surface. In controls (CTRL) and in cells cultivated with VitD alone, TF was almost not detectable on membranes (0.1%). Stimulation with IL-6 caused TF+ in 9.8 ± 1.8% of cells. In IL-6-treated cells preincubated with VitD, TF+ cells were reduced to 1.2 ± 0.8%. LPS served as positive control. (**B**) Graph of FACS analysis experiment. Each point represents the mean ± SD of 6 experiments. (up to 18% TF^+^ cells; * *p* < 0.001 vs. Control; ** *p* < 0.001 vs. IL-6 with Tukey’s post hoc test). (**C**) Effects of VitD on IL-6-induced TF activity evaluated by a two-step colorimetric assay based on the ability of TF/FVIIa to promote generation of coagulation FXa. IL-6-induced-TF activity reflects results observed for TF expression, confirming that TF was functionally active. VitD preincubation significantly reduces TF activity. The use of ZK15 abolished the VitD effect. LPS served as positive control. Each column represents the mean ± SD of 6 experiments in triplicate (* *p* < 0.001 vs. Control; ** *p* < 0.001 vs. IL-6, ^#^ *p* = NS vs. IL-6; one-way ANOVA with Tukey’s post hoc test).

Tissue Factor expression on cell surface was measured by FACS analysis. TF was almost undetectable on unstimulated HUVECs, at baseline as well as in cells stimulated with VitD alone. Incubation with IL-6 caused a significant increase of TF expression on the HUVEC surface (Figure 2A,B). Similarly, TF procoagulant activity, determined by a two-step colorimetric assay, based on the ability of TF to promote generation of coagulation FXa, was almost undetectable at baseline and on cells stimulated with VitD. The procoagulant activity increased after stimulation with IL-6 (Figure 2C). VitD prevented the effects exerted by IL-6 on TF expression and activity (Figure 2). Moreover, in the presence of ZK15, TF procoagulant activity was comparable to the IL-6 effect (Figure 2C).

### 3.2. ACE2 Receptor mRNA and Protein Expression in Vitamin D Pretreated Cells

**Figure 3:**
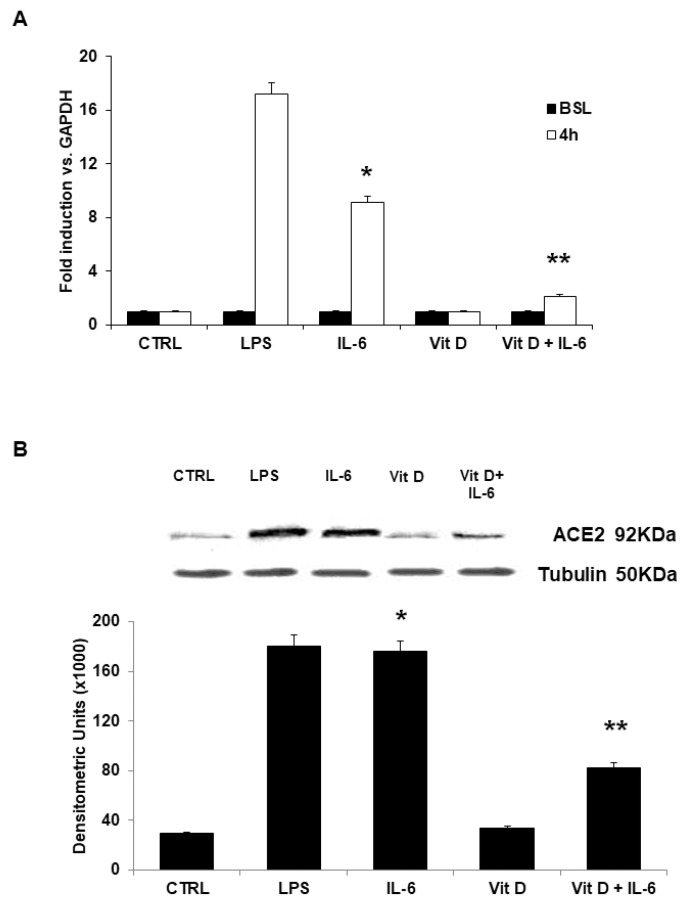
(**A**) Effects of VitD on IL-6-induced ACE2r transcription in human endothelial cells assessed by Real Time quantitative PCR. ACE2r mRNA was undetectable at baseline. Incubation with IL-6 caused significant increase in ACE2r mRNA levels, as compared to unstimulated cells. Preincubation with VitD inhibited the IL-6 effect on ACE2r-mRNA. LPS served as positive control. Data are expressed as fold induction versus control gene represented by GAPDH. Each bar represents the mean ± SD of 3 different experiments. (* *p* < 0.001 vs. Control; ** *p* < 0.001 vs. oxLDL; one-way ANOVA with Tukey’s post hoc test). (**B**) Effects of VitD on IL-6-induced ACE2r protein evaluated by Western blot analysis of cell lysates. IL-6 caused a significant increase of ACE2r protein levels. These effects were prevented by treatment with VitD. Tubulin served as loading control. LPS served as positive control. Each bar represents the mean ± SD of 6 different experiments (* *p* < 0.001 vs. Control; ** *p* < 0.001 vs. IL-6; one-way ANOVA with Tukey’s post hoc test). The insert shows results of a representative experiment.

Levels of mRNA for ACE2r were examined by Real time PCR. In unstimulated cells, levels of ACE2r-mRNA were very low. Incubation with VitD alone did not cause any variation in gene levels. On the contrary, IL-6 stimulation resulted in a significant induction of ACE2r-mRNA levels as compared to unstimulated cells. These IL-6 effects on mRNA levels were significantly reduced in HUVECs incubated with VitD. (Figure 3A). Similarly, very low levels of protein were detected in unstimulated cells and in HUVEC cultivated with VitD (Figure 3B). Levels of ACE2r protein significantly increased in cells stimulated with IL-6. These effects were significantly reduced in HUVECs incubated with VitD (Figure 3B).

### 3.3. Vitamin D Reduces Adhesion Molecules’ Expression

**Figure 4:**
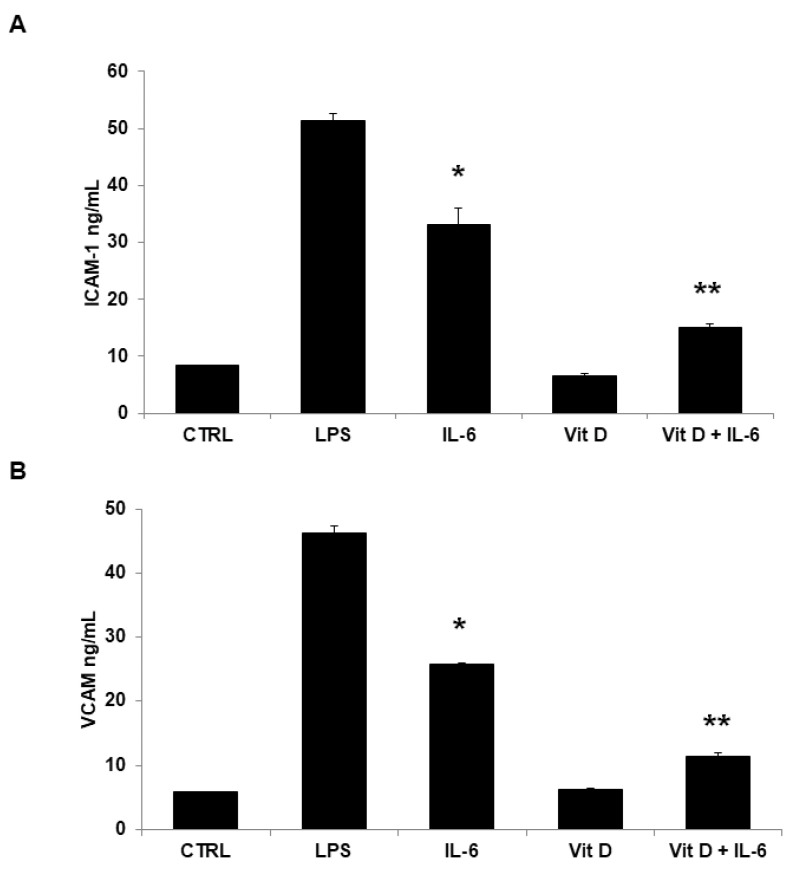
In basal conditions, very low basal levels of soluble ICAM-1 and VCAM-1 were measured in the HUVEC supernatants. Stimulation with IL-6 increased significantly the supernatant concentrations: ICAM-1 ((**A**) 33.16 ± 2.75 ng/mL, * *p* < 0.001 vs. Control; one-way ANOVA with Tukey’s post hoc test) and VCAM-1 ((**B**) 25.83 ± 0.21 ng/mL, * *p* < 0.001 vs. Control; one-way ANOVA with Tukey’s post hoc test). VitD preincubation significantly prevented both soluble ICAM-1 ((**A**) 15.06 ± 0.7 ng/mL, ** *p* < 0.001 vs. IL-6-treated ECs; one-way ANOVA with Tukey’s post hoc test) and VCAM-1 levels ((**B**) 11.39 ± 0.62 ng/mL, ** *p* < 0.001 vs. IL-6-treated ECs; one-way ANOVA with Tukey’s post hoc test). Each column represents the mean ± SD of 6 different experiments.

Soluble CAMs were assessed in cell supernatants by specific ELISA kits. Very low basal levels of soluble ICAM-1 and VCAM-1 were measured in unstimulated cells and in cells stimulated with VitD alone. On the contrary, incubation with IL-6 was associated with a significant increase of soluble CAM levels in cell supernatants. VitD preincubation significantly prevented ICAM-1 and VCAM-1 expression at a similar extent (Figure 4A,B).

### 3.4. Regulation of Intracellular Signaling: The Role of NF-κB Nuclear Translocation and STAT3

**Figure 5:**
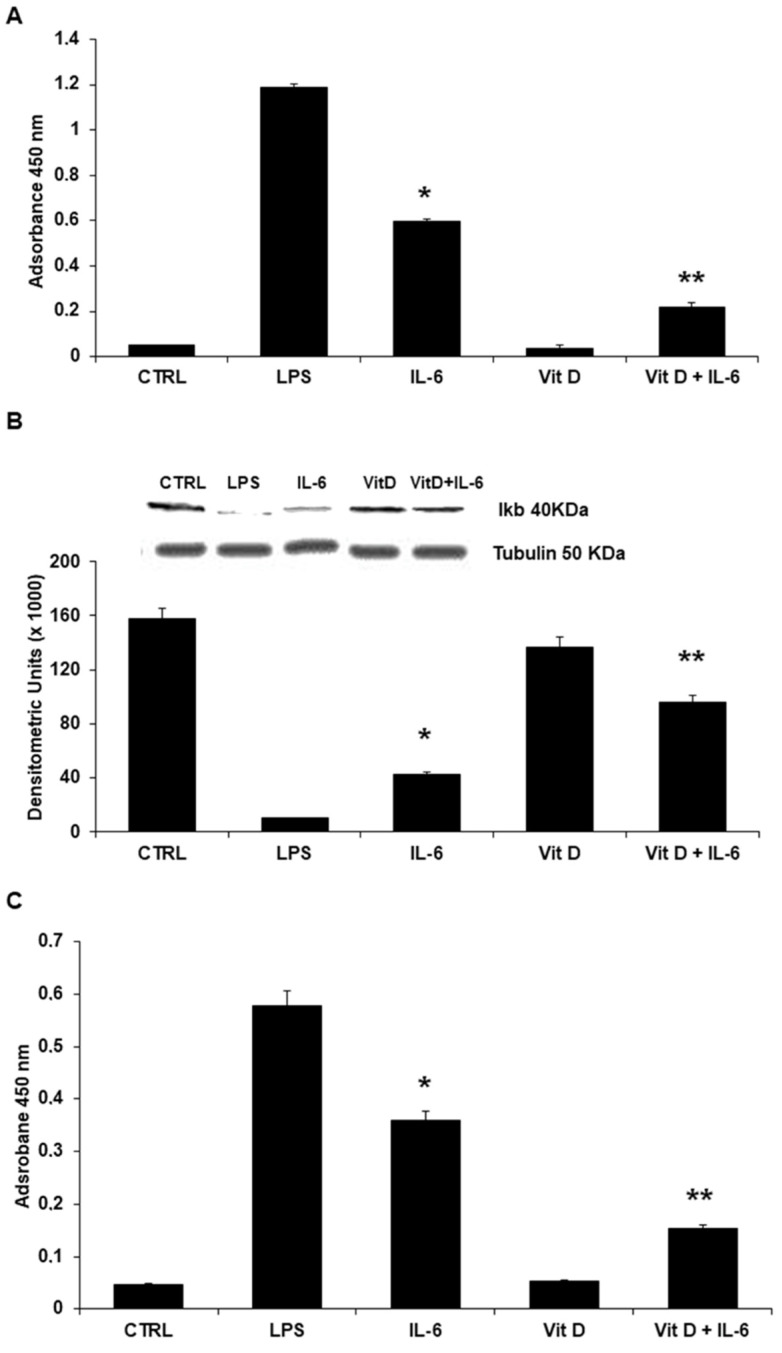
(**A**) In HUVEC incubated with IL-6, a significant increase of NF-kB levels was observed. Pretreatment of endothelial cells with VitD prevented the IL-6 effects on NF-kB. LPS served as positive control. Each bar represents the mean ± SD of 3 different experiments. (* *p* < 0.001 vs. Control; ** *p* < 0.001 vs. IL-6, one-way ANOVA with Tukey’s post hoc test). (**B**) Western blot analyses. IL-6 stimulation results in significant reduced levels of IκB. Conversely, preincubation with VitD restores IκB levels. LPS served as positive control. Each bar represents the mean ± SD of 3 different experiments. (* *p* < 0.001 vs. Control; ** *p* < 0.001 vs. IL-6, one-way ANOVA with Tukey’s post hoc test). The insert shows results of a representative experiment. (**C**) In HUVEC incubated with IL-6, a significant increase of STAT3 levels was observed. Pretreatment of endothelial cells with VitD prevented the IL-6 effects on STAT3. LPS served as positive control. Each bar represents the mean ± SD of 3 different experiments. (* *p* < 0.001 vs. Control; ** *p* < 0.001 vs. IL-6, one-way ANOVA with Tukey’s post hoc test).

Nuclear translocation of NF-κB was assessed by a non-radioactive, sensitive method for detecting the specific transcription factor DNA binding activity. NF-κB translocation was not observed in unstimulated cells and in cells stimulated with VitD alone. In cells preincubated with VitD before being stimulated with IL-6, NF-κB translocation from the cytosol to the nucleus was significantly reduced (Figure 5A). As expected, and in line with results of NF-κB translocation, IκB cytoplasmatic levels evaluated by Western blot were reduced in HUVECs stimulated with IL-6 and normalized in cells preincubated with VitD (Figure 5B). Activation of STAT3 was observed in IL-6-treated cells. This phenomenon was significantly reduced in cells incubated with VitD (Figure 5C).

### 3.5. Modulation of NLRP3 Protein Expression

**Figure 6:**
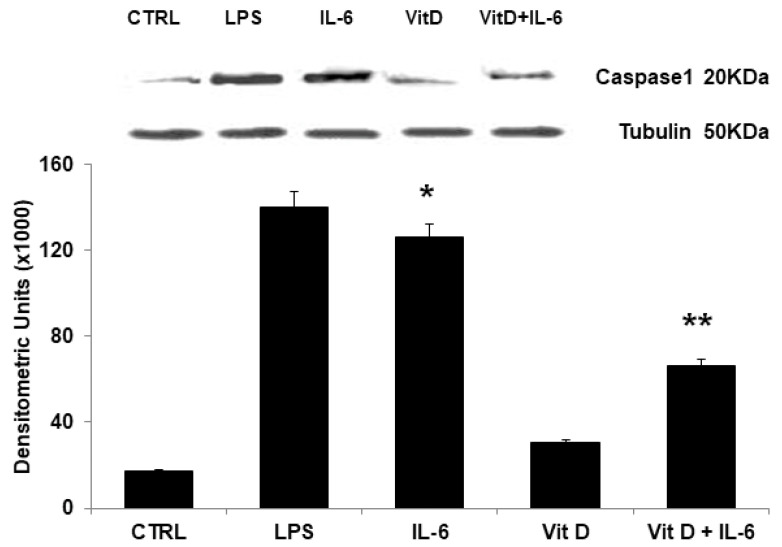
IL-6 caused a significant increase in Caspase-1 protein levels. These effects were prevented by treatment with VitD. Tubulin served as loading control. LPS served as positive control. Each bar represents the mean ± SD of 6 different experiments (* *p* < 0.001 vs. Control; ** *p* < 0.001 vs. IL-6; one-way ANOVA with Tukey’s post hoc test). The insert shows results of a representative experiment.

To evaluate the activation of NLRP3 inflammasome, caspase-1 expression was evaluated by Western blot. Caspase-1 was almost undetectable in unstimulated cells and in cells stimulated with VitD. IL-6 caused significant increase of caspase-1 expression. VitD pretreatment significantly reduced IL-6 effects (Figure 6).

## 4. Discussion

The most important findings of the present study are: (1) IL-6 exerts prothrombotic effects on human endothelial cells because it promotes expression of the TF gene and TF protein with procoagulant activity, (2) IL-6 increases levels of soluble adhesion molecules, NLRP3 expression as well as ACE2r protein, suggesting that these might be potential mediators modulating the observed COVID-19 endothelial effects, (3) VitD significantly reduces these effects via modulation of NF-κB and STAT3 activation.

Much evidence has shown the close relationship between inflammation and thrombosis in several diseases [1]. For example, the pathophysiology of atherosclerosis and of its thrombotic complications recognizes an important role for immune-inflammation [24]. Moreover, inflammation is associated with thrombotic events in the recent COVID-19 infection [6,11,12,25,26]. In both these diseases, endothelial dysfunction seems to play a pivotal role in the pathophysiology of the observed inflammation-associated thrombosis [3,27,28]. Specifically, in the COVID-19 infection, the viral attack to the host causes a maladaptive immunological response that increases vascular permeability, finally leading to endothelial dysfunction and activation of coagulation [29,30]. In this host response, which involves several immunological cells and the release of several cytokines, COVID-19 infection is associated with a cytokine storm in which IL-6 levels are strongly increased, especially in those critical patients in which thrombosis is enhanced [11,31]. Here, we have demonstrated that IL-6 exerts prothrombotic effects on human endothelial cells because it promotes expression of functional TF, suggesting that this might be one of the possible mechanisms by which COVID-19 causes thrombosis. Another suggested mechanism of disease for COVID-19 is the loss of the endothelial integrity associated with changes in the surface expression of intracellular adhesion molecule-1 (ICAM1), vascular cell adhesion protein-1 (VCAM1) and the tight junction scaffold protein zonula occludens-1 (ZO-1) [32]. In this report we have demonstrated that IL-6 increases soluble levels of adhesion molecules suggesting that this might be one of the chemical mediators modulating the observed COVID-19 endothelial effects.

The novel coronavirus infects its target cells by binding to the angiotensin-converting enzyme 2 receptor (ACE2r), which is the main receptor for COVID-19 [33]. The same ACE2r has been shown as the entry door for COVID-19 in endothelial cells too, finally leading to impaired endothelial homeostasis [8]. Interestingly, we have found that IL-6 increases gene as well as protein expression of ACE2r in endothelial cells, suggesting that this might be one of the hypothetical mechanisms by which the virus amplifies its action on endothelial cells.

It has been reported that NF-κB activation is one of the main molecular mechanism(s) involved in the acute respiratory RNA virus-induced cytokine storm [12,34]. Specifically, binding of COVID-19 to ACE2r triggers receptor endocytosis into the host cell. As result, several intracellular events occur that start with the activation of the Toll-like receptors (TLRs), continue with the translocation of the NF-κB into the nucleus and end with transcription of several genes coding for pro-inflammatory proteins, including IL-6 [12,34]. Up-regulated expression of the IL-6 gene is followed by increased levels in the plasma of this cytokine that binds to its receptor and leads to activation of the STAT3 pathway. This pathway, together with the NF-kB pathway, causes a cytokine release syndrome with a complex multi-inflammatory response [21]. Of note, previous reports have demonstrated that NF-κB might be actively involved in the pathophysiology of vascular thrombotic events since binding sites for this transcription factor are contained in the promoter for TF, the key activator of extrinsic coagulation pathway [19,20,35]. Thus, it is conceivable that COVID-19 might promote thrombotic events by this NF-kB mediated pathway. In line with previous observations [31], in the present report we show that IL-6, one of the key cytokines of the COVID inflammatory storm, increases translocation of NF-κB to the nucleus as well as activation of STAT3. It is known that the induction of such an inflammatory process in the host cell is often associated the engagement of inflammasomes, which are protein platforms that aggregate in the cytosol in response to different stimuli [36]. The most studied protein of these platforms is the NLRP3 inflammasome that contains Caspase-1. Inflammasome activation in response to COVID-19 infection and its association with a worst prognosis in COVID-19 patients has been reported [37]. In line with these reports, we have demonstrated that IL-6 increased inflammasome levels in endothelial cells.

Vitamin D is a fat-soluble vitamin [38] that seems to have a protective role in several diseases and especially in cardiovascular disease such as hypertension, myocardial infarction, heart failure and stroke [13]. Several mechanisms have been proposed to explain the potential benefits of VitD, such as modulation of genes involved in cell proliferation and differentiation, apoptosis, oxidative stress, membrane transport, matrix homeostasis and cell adhesion [39]. Controversial results have been published on the role of VitD in the COVID-19 pandemic scenario. Some data have pointed out that plasma levels of VitD might be closely related to severity of this disease [17]. Recent data from 20 different European countries showed a negative correlation between VitD levels and the number of cases of COVID-19/1 million population in each country [40]. Another report indicates that VitD deficiency is markedly associated with a more severe COVID-19 infection, higher inflammatory response, and increased morbidity and mortality [41]. On the other hand, a more recent meta-analysis did not link VitD deficiency or insufficiency to susceptibility to COVID-19 infection or death [42]. The large heterogeneity of the available studies seems to not allow establishment of a definitive cause–effect relationship [43].

Again, VitD has been indicated as an important regulator of immune response [44,45] that is able to exert antiviral effects too [46,47]. For example, in macrophage, VitD has shown a predominant impact on cytokine response beyond viral killing [45]. Finally, in different experimental models of pneumonia and pneumonitis, VitD has had a consistent role in reducing the inflammatory cytokine response to pathogens [48,49]. Beyond these immune-inflammatory effects, other reports have clearly indicated that VitD is also involved in the regulation of thrombotic pathways, since its deficiency has been reported to be associated with an increased risk of thrombotic events [50]. In the present report we show that VitD significantly reduces the effects of IL-6 on TF gene and protein expression. Importantly, this vitamin inhibited IL-6-induced TF procoagulant activity on the cell surface. In line with our previous report [19], the effect of VitD seems to be mediated by the VitD receptor (VDR) since the use of ZK15, a specific VDR antagonist, abolished the potential benefit on TF expression and activity exerted by VitD.

## 5. Conclusions

Results of the present study, with all potential limitations derived from an in vitro study, suggest that IL-6 might play a pivotal role in the potential pathophysiological scenario in which COVID-19 causes thrombosis. Specifically, we showed that IL-6, one of the main chemical mediators involved in the cytokine storm for COVID-19 infection, induces expression of gene and of functionally active Tissue Factor in human endothelial cells, shifting them to a pro-thrombotic phenotype. Moreover, this cytokine induces expression of the soluble adhesion molecules ICAM-1 and VCAM-1, markers of endothelial dysfunction. IL-6 seems to exert these effects by modulating the NF-kB as well as the STAT3 pathways. Moreover, IL-6 up-regulates levels of ACE2, the natural receptor of COVID-19, finally amplifying the virus effects on endothelial cells. Interestingly, we showed that VitD is able to prevent all these IL-6 deleterious effects by modulating the activity of the transcription factor NF-κB and of STAT3. Taken together, data of the present study shed a brighter light on the still partially unresolved issue about VitD beneficial effects observed in patients with IL-6-related inflammation such as COVID-19 infection, suggesting a possible molecular mechanism by which this vitamin exerts its action. However, rigorous and well-conducted clinical trials are needed to address this issue in the clinical scenario.

## References

[1] Jackson S.P., Darbousset R., Schoenwaelder S.M. (2019). Thromboinflammation: Challenges of therapeutically targeting coagulation and other host defense mechanisms. Blood.

[2] Ma R.C.J., Voetsch B., Loscalzo J. (2005). Endogenous mechanisms of inhibition of platelet function. Microcirculation.

[3] Gimbrone M.A., Garcia-Cardeña G. (2016). Endothelial Cell Dysfunction and the Pathobiology of Atherosclerosis. Circ. Res..

[4] Galkina E., Ley K. (2007). Vascular adhesion molecules in atherosclerosis. Arterioscler. Thromb. Vasc. Biol..

[5] Grover S.P., Mackman N. (2018). Tissue Factor: An Essential Mediator of Hemostasis and Trigger of Thrombosis. Arterioscler. Thromb. Vasc. Biol..

[6] Labò N., Ohnuki H., Tosato G. (2020). Vasculopathy and Coagulopathy Associated with SARS-CoV-2 Infection. Cells.

[7] Godino C., Scotti A., Maugeri N., Mancini N., Fominskiy E., Margonato A., Landoni G. (2021). Antithrombotic therapy in patients with COVID-19?—Rationale and Evidence. Int. J. Cardiol..

[8] Shang J., Wan Y., Luo C., Ye G., Geng Q., Auerbach A., Li F. (2020). Cell entry mechanisms of SARS-CoV-2. Proc. Natl. Acad. Sci. USA.

[9] Zhou P., Yang X.-L., Wang X.-G., Hu B., Zhang L., Zhang W., Si H.-R., Zhu Y., Li B., Huang C.-L. (2020). A pneumonia outbreak associated with a new coronavirus of probable bat origin. Nature.

[10] Fajgenbaum D.C., June C.H. (2020). Cytokine Storm. N. Engl. J. Med..

[11] Del Valle D.M., Kim-Schulze S., Huang H.-H., Beckmann N.D., Nirenberg S., Wang B., Lavin Y., Swartz T.H., Madduri D., Stock A. (2020). An inflammatory cytokine signature predicts COVID-19 severity and survival. Nat. Med..

[12] Hojyo S., Uchida M., Tanaka K., Hasebe R., Tanaka Y., Murakami M., Hirano T. (2020). How COVID-19 induces cytokine storm with high mortality. Inflamm. Regen..

[13] Kouvari M., Panagiotakos D.B. (2019). Vitamin D status, gender and cardiovascular diseases: A systematic review of prospective epidemiological studies. Expert Rev. Cardiovasc. Ther..

[14] Wimalawansa S.J. (2018). Vitamin D and cardiovascular diseases: Causality. J. Steroid Biochem. Mol. Biol..

[15] Schwarz N., Nicholls S.J., Psaltis P.J. (2018). Vitamin D and Cardiovascular Disease. Heart Lung Circ..

[16] Martineau A.R., Jolliffe D.A., Hooper R.L., Greenberg L., Aloia J.F., Bergman P., Dubnov-Raz G., Esposito S., Ganmaa D., Ginde A.A. (2017). Vitamin D supplementation to prevent acute respiratory tract infections: Systematic review and meta-analysis of individual participant data. BMJ.

[17] Laird E., Rhodes J., Kenny R.A. (2020). Vitamin D and Inflammation: Potential Implications for Severity of COVID-19. Ir. Med. J..

[18] Martinez-Moreno J.M., Herencia C., Montes de Oca A., Munoz-Castaneda J.R., Rodriguez-Ortiz M.E., Diaz-Tocados J.M., Peralbo-Santaella E., Camargo A., Canalejo A., Rodriguez M. (2016). Vitamin D modulates tissue factor and protease-activated receptor 2 expression in vascular smooth muscle cells. FASEB J..

[19] Cimmino G., Morello A., Conte S., Pellegrino G., Marra L., Golino P., Cirillo P. (2020). Vitamin D inhibits Tissue Factor and CAMs expression in oxidized low-density lipoproteins-treated human endothelial cells by modulating NF-kappaB pathway. Eur. J. Pharmacol..

[20] Cirillo P., Conte S., Cimmino G., Pellegrino G., Ziviello F., Barra G., Sasso F.C., Borgia F., De Palma R., Trimarco B. (2017). Nobiletin inhibits oxidized-LDL mediated expression of Tissue Factor in human endothelial cells through inhibition of NF-kappaB. Biochem. Pharmacol..

[21] Luo Y., Zheng S.G. (2016). Hall of Fame among Pro-inflammatory Cytokines: Interleukin-6 Gene and Its Transcriptional Regulation Mechanisms. Front. Immunol..

[22] Freeman T.L., Swartz T.H. (2020). Targeting the NLRP3 Inflammasome in Severe COVID-19. Front. Immunol..

[23] Ratajczak M.Z., Kucia M. (2020). SARS-CoV-2 infection and overactivation of Nlrp3 inflammasome as a trigger of cytokine “storm” and risk factor for damage of hematopoietic stem cells. Leukemia.

[24] Cimmino G., Loffredo F.S., Morello A., D’Elia S., De Palma R., Cirillo P., Golino P. (2017). Immune-Inflammatory Activation in Acute Coronary Syndromes: A Look into the Heart of Unstable Coronary Plaque. Curr. Cardiol. Rev..

[25] Raggi P., Genest J., Giles J.T., Rayner K.J., Dwivedi G., Beanlands R.S., Gupta M. (2018). Role of inflammation in the pathogenesis of atherosclerosis and therapeutic interventions. Atherosclerosis.

[26] Abou-Ismail M.Y., Diamond A., Kapoor S., Arafah Y., Nayak L. (2020). The hypercoagulable state in COVID-19: Incidence, pathophysiology, and management. Thromb. Res..

[27] Gladka M.M., Maack C. (2020). The endothelium as Achilles’ heel in COVID-19 patients. Cardiovasc. Res..

[28] Libby P., Lüscher T. (2020). COVID-19 is, in the end, an endothelial disease. Eur. Heart J..

[29] Goeijenbier M., van Wissen M., van de Weg C., Jong E., Gerdes V.E., Meijers J.C., Brandjes D.P., van Gorp E.C. (2012). Review: Viral infections and mechanisms of thrombosis and bleeding. J. Med. Virol..

[30] Rajendran P., Rengarajan T., Thangavel J., Nishigaki Y., Sakthisekaran D., Sethi G., Nishigaki I. (2013). The vascular endothelium and human diseases. Int. J. Biol. Sci..

[31] Giannakodimos I., Gkountana G.-V., Lykouras D., Karkoulias K., Tsakas S. (2021). The role of Interleukin-6 in the pathogenesis, prognosis and treatment of severe COVID-19. Curr. Med. Chem..

[32] Buzhdygan T.P., DeOre B.J., Baldwin-Leclair A., McGary H., Razmpour R., Galie P.A., Potula R., Andrews A.M., Ramirez S.H. (2020). The SARS-CoV-2 spike protein alters barrier function in 2D static and 3D microfluidic in vitro models of the human blood-brain barrier. bioRxiv.

[33] Hamming I., Timens W., Bulthuis M.L., Lely A.T., Navis G., van Goor H. (2004). Tissue distribution of ACE2 protein, the functional receptor for SARS coronavirus. A first step in understanding SARS pathogenesis. J. Pathol..

[34] Kircheis R., Haasbach E., Lueftenegger D., Heyken W.T., Ocker M., Planz O. (2020). NF-kappaB Pathway as a Potential Target for Treatment of Critical Stage COVID-19 Patients. Front. Immunol..

[35] Cirillo P., Ziviello F., Pellegrino G., Conte S., Cimmino G., Giaquinto A., Pacifico F., Leonardi A., Golino P., Trimarco B. (2015). The adipokine apelin-13 induces expression of prothrombotic tissue factor. Thromb. Haemost..

[36] Broz P., Dixit V.M. (2016). Inflammasomes: Mechanism of assembly, regulation and signalling. Nat. Rev. Immunol..

[37] Rodrigues T.S., de Sá K.S.G., Ishimoto A.Y., Becerra A., Oliveira S., Almeida L., Gonçalves A.V., Perucello D.B., Andrade W.A., Castro R. (2021). Inflammasomes are activated in response to SARS-CoV-2 infection and are associated with COVID-19 severity in patients. J. Exp. Med..

[38] Bikle D.D. (2014). Vitamin D metabolism, mechanism of action, and clinical applications. Chem. Biol..

[39] Norman P.E., Powell J.T. (2014). Vitamin D and cardiovascular disease. Circ. Res..

[40] Ilie P.C., Stefanescu S., Smith L. (2020). The role of vitamin D in the prevention of coronavirus disease 2019 infection and mortality. Aging Clin. Exp. Res..

[41] Jain A., Chaurasia R., Sengar N.S., Singh M., Mahor S., Narain S. (2020). Analysis of vitamin D level among asymptomatic and critically ill COVID-19 patients and its correlation with inflammatory markers. Sci. Rep..

[42] Chen J., Mei K., Xie L., Yuan P., Ma J., Yu P., Zhu W., Zheng C., Liu X. (2021). Low vitamin D levels do not aggravate COVID-19 risk or death, and vitamin D supplementation does not improve outcomes in hospitalized patients with COVID-19: A meta-analysis and GRADE assessment of cohort studies and RCTs. Nutr. J..

[43] Crafa A., Cannarella R., Condorelli R.A., Mongioì L.M., Barbagallo F., Aversa A., La Vignera S., Calogero A.E. (2021). Influence of 25-hydroxy-cholecalciferol levels on SARS-CoV-2 infection and COVID-19 severity: A systematic review and meta-analysis. EClinicalMedicine.

[44] Bilezikian J.P., Bikle D., Hewison M., Lazaretti-Castro M., Formenti A.M., Gupta A., Madhavan M.V., Nair N., Babalyan V., Hutchings N. (2020). MECHANISMS IN ENDOCRINOLOGY: Vitamin D and COVID-19. Eur. J. Endocrinol..

[45] Zdrenghea M.T., Makrinioti H., Bagacean C., Bush A., Johnston S.L., Stanciu L.A. (2017). Vitamin D modulation of innate immune responses to respiratory viral infections. Rev. Med. Virol..

[46] Telcian A.G., Zdrenghea M.T., Edwards M.R., Laza-Stanca V., Mallia P., Johnston S.L., Stanciu L.A. (2017). Vitamin D increases the antiviral activity of bronchial epithelial cells in vitro. Antivir. Res..

[47] Schögler A., Muster R.J., Kieninger E., Casaulta C., Tapparel C., Jung A., Moeller A., Geiser T., Regamey N., Alves M.P. (2016). Vitamin D represses rhinovirus replication in cystic fibrosis cells by inducing LL-37. Eur. Respir. J..

[48] Zhang Y., Leung D.Y., Richers B.N., Liu Y., Remigio L.K., Riches D.W., Goleva E. (2012). Vitamin D inhibits monocyte/macrophage proinflammatory cytokine production by targeting MAPK phosphatase-1. J. Immunol..

[49] Tsujino I., Ushikoshi-Nakayama R., Yamazaki T., Matsumoto N., Saito I. (2019). Pulmonary activation of vitamin D3 and preventive effect against interstitial pneumonia. J. Clin. Biochem. Nutr..

[50] Mohammad S., Mishra A., Ashraf M.Z. (2019). Emerging Role of Vitamin D and its Associated Molecules in Pathways Related to Pathogenesis of Thrombosis. Biomolecules.

